# Outcomes of Idecabtagene Vicleucel Therapy in Patients with Relapsed/Refractory Multiple Myeloma: A Single-Institution Experience

**DOI:** 10.3390/biomedicines13010036

**Published:** 2024-12-27

**Authors:** Aaron Trando, Farid Ghamsari, Philip Yeung, Caitlin Costello, Ila Saunders, Ah-Reum Jeong

**Affiliations:** 1 School of Medicine, University of California San Diego, La Jolla, CA 92093, USA; atrando@health.ucsd.edu; 2 Department of Internal Medicine, University of California San Diego, La Jolla, CA 92093, USA; 3 Master of Advanced Studies (MAS) Program in Clinical Research, University of California San Diego, La Jolla, CA 92093, USA; 4 Department of Medicine, Division of Blood and Marrow Transplantation, University of California San Diego, La Jolla, CA 92093, USA; 5 Skaggs School of Pharmacy & Pharmaceutical Sciences, University of California San Diego, La Jolla, CA 92093, USA; isaunders@health.ucsd.edu

**Keywords:** CAR T-cell therapy, ide-cel, multiple myeloma, CRS, ICANS, cytomegalovirus

## Abstract

**Background/Objectives:** Idecabtagene vicleucel (ide-cel), an anti-B-cell maturation chimeric antigen receptor T-cell therapy, represents an unprecedented treatment option for relapsed/refractory multiple myeloma (R/R MM). Nevertheless, given its limitations, including the risk of adverse effects and unclear durability of efficacy, there remains a need to report the real-world clinical outcomes of ide-cel therapy in patients with R/R MM, as well as explore host predictive factors for therapy. **Methods:** We performed a single-center retrospective analysis of 25 adult patients with R/R MM who received ide-cel between 2021 and 2023 at the University of California San Diego Health. Data on baseline characteristics, efficacy, safety, and post-relapse outcomes were collected. Treatment responses were assessed using the International Myeloma Working Group criteria while survival analyses were conducted using the Kaplan–Meier and Cox proportional hazards methods. **Results:** The median age was 65. Twelve patients (48%) were male. Patients received a median of six lines of prior therapy with four patients (16%) receiving prior BCMA-targeted therapy. Six patients (24%) had high-risk cytogenetics while ten patients (40%) had extramedullary disease. The incidence of cytokine release syndrome and immune effector cell-associated neurotoxicity syndrome incidence was 92% and 12%, respectively. All grade infection occurred in 11 patients (44%). Cytomegalovirus (CMV) reactivation occurred in 9 of 19 patients (47%) who were CMV IgG positive prior to CAR T-cell therapy. The objective response rate (ORR) was 84%; stringent complete response was seen in 14 patients (56%). After a median follow-up of 13 months, median progression-free survival (PFS) was 13.9 months (95% CI: 9.21 months—not reached [NR]); median overall survival (OS) was not reached (95% CI: 19.5 months—NR). Among the 11 patients (44%) who progressed after ide-cel therapy, median OS2 was 13.7 months; especially poor outcomes (median OS2 of 1.74 months) were observed in four patients who did not respond to ide-cel. Six of these eleven patients remained alive at time of data cutoff. Univariate and multivariate analysis revealed no significant predictors of ORR, PFS, or OS. **Conclusions:** Overall, ide-cel had comparable efficacy and safety to the KarMMa-1 trial and other reported real-world experiences.

## 1. Introduction

Since its inception over 30 years ago, chimeric antigen receptor T-cell (CAR T-cell) therapy has achieved groundbreaking success in treating different hematologic malignancies. There are currently six CAR T-cell products that have been approved by the US Food and Drug Administration (FDA) for treatment of a wide spectrum of hematologic malignancies, from acute lymphoblastic leukemia to multiple subtypes of B-cell non-Hodgkin lymphoma [1,2].

Idecabtagene vicleucel (ide-cel) is a novel CAR T-cell therapy that targets B-cell maturation antigen (BCMA), which is selectively expressed on mature B lymphocytes and has a relevant role for their survival and proliferation, explaining its value as a therapeutic target for R/R MM [3]. The ide-cel construct itself is comprised of an extracellular BCMA-detecting murine single chain variable fragment, a human CD8 alpha hinge, and an intracellular domain that consists of a costimulatory 4-1BB molecule attached to a CD3 zeta T-cell activation domain [4]. Data from the phase 2 KarMMa-1 trial yielded an objective response rate (ORR) of 73% and a complete response rate (CRR) of 33% among 128 patients with R/R MM who were treated with ide-cel [5]. Given the promising results of this trial, the FDA approved ide-cel in March 2021 for patients with R/R MM who have failed at least four prior lines of therapy [6], including a proteasome inhibitor (PI), immunomodulatory drug (IMID), and anti-CD38 monoclonal antibody (mAb).

Through 2022, ide-cel appeared to provide a clinical benefit for many patients with R/R MM, especially those with triple-class refractory disease (refractory to a PI, IMID, and a CD38 mAb). However, there remain barriers to effectively utilizing ide-cel across the entirety of the R/R MM patient population. First, while ide-cel is generally associated with a manageable tolerability profile, like other CAR-T cell products, it still carries the serious risks of cytokine release syndrome (CRS) and immune effector cell–associated neurotoxicity syndrome (ICANS) [4]. Furthermore, based on large-scale clinical trial data from KarMMa-1, median progression-free survival (PFS) for patients receiving ide-cel was only 8.8 months [5]. Within this group of patients who fail to respond or do not maintain a response after treatment with ide-cel, survival outcomes have been reported to be especially poor [7].

There remain numerous clinical issues regarding how to improve the application of ide-cel therapy in the R/R MM patient population. First, biomarkers of ide-cel outcomes, including those that predict the onset of toxicity and overall response to therapy, are lacking [8]. At the same time, the incidence and management of key complications of ide-cel therapy, such as cytomegalovirus (CMV) reactivation, have not been clearly elucidated in the literature [9]. Finally, standardized treatment approaches for patients who progress/relapse after ide-cel are not currently well defined, either [10]. Thus, we conducted a single-center retrospective study of patients with R/R MM who received ide-cel to share our real-world experience with using this specific anti-BCMA agent, including safety, efficacy, and post-relapse outcomes.

## 2. Materials and Methods

### 2.1. Study Population

This single-institution retrospective review consisted of patients with R/R MM who received ide-cel at the University of California of San Diego (UCSD) between October 2021 and July 2023; date of data censorship was 1 January 2024. There were no specific exclusion criteria (including baseline kidney function and extramedullary disease involvement) that precluded patients from receiving ide-cel, as the clinical judgment of each treating physician was used to assess patient candidacy for ide-cel. The study received approval from the UCSD Institutional Review Board and was conducted per institutional guidelines and the principles of the Declaration of Helsinki.

### 2.2. Data Extraction

For all eligible patients, a complete retrospective review of the electronic medical records was conducted. Data extracted included baseline characteristics/labs, as well as safety/efficacy outcomes. Pathology data (including cytogenetics results) were obtained both at the time of initial diagnosis and at the time of CAR T-cell therapy. High-risk cytogenetics was defined as having one or more of t(4;14), t(14;16), deletion 17p/p53 mutation/monosomy 17, or gain/amp of 1q. Double-hit disease was defined as disease possessing any two high-risk cytogenetic abnormalities [11]. Triple-class refractory disease was defined as disease refractory to at least one each of an IMID, PI, and anti-CD38 mAb. Penta-refractory disease was defined as disease refractory to lenalidomide, pomalidomide, bortezomib, carfilzomib, and daratumumab [12].

Safety outcomes consisted of CRS and ICANS incidences, which were graded per the American Society for Transplantation and Cellular Therapy guidelines [13]. Additionally, cytopenias and infectious complications were evaluated and graded within the first 6 months of ide-cel infusion using the Common Terminology Criteria for Adverse Events v5.0 criteria [14]. Treatment responses were assigned according to the International Myeloma Working Group criteria [12]. ORR was defined as patients who achieved one of the following as their best response to ide-cel therapy: stringent complete response (sCR), complete response (CR), very good partial response (VGPR), or partial response (PR). Data regarding post-relapse treatment regimens and outcomes were only recorded for patients who ended up relapsing or being refractory to ide-cel therapy [12].

### 2.3. Statistical Analysis

In order to outline patient characteristics and other baseline variables, descriptive statistics were employed. Meanwhile, to compare dichotomous and continuous variables between groups of patients, chi-square tests, *t*-tests, correlation matrices, and other non-parametric tests were run as appropriate for the distribution of variables.

Clinically relevant endpoints were defined as the following: progression-free survival (PFS)—date of ide-cel infusion to time of progression, relapse, or death; overall survival (OS)—date of ide-cel infusion to time of death; OS2—time from relapse/progression after CAR T-cell therapy to death. The Kaplan–Meier (KM) method was used to calculate the survival probability of PFS. The reverse KM method was used to determine median follow-up time. In evaluating predictive markers of outcomes, univariate Cox regression models were performed for high-risk prognostic factors. A multiple multivariable Cox regression model embedded with a backward selection method was then employed to identify significant covariates at a cutoff of 0.05. A similar process was applied to assess the logistic regression of patients’ best overall responses (defined as the aggregate of sCR, CR, VGPR, and PR). A *p*-value of <0.05 was considered to be statistically significant for all statistical analyses.

R studio version 2023.03.1.3 was used to carry out all statistical analyses.

## 3. Results

### 3.1. Baseline Patient Characteristics

A total of 25 patients with R/R MM were included, all of whom received ide-cel. At the time of CAR T-cell infusion (day 0), median age was 65 years (range, 50–79). Six patients (24%) were over the age of 70, and twelve patients (48%) were male (Table 1).

The revised International Staging System (R-ISS) score at the time of diagnosis was unknown for the majority of the patients. Twelve of twenty-five patients (48%) had high-risk cytogenetic features any time prior to receiving ide-cel therapy. Two patients (10%) had two or more high-risk cytogenetic abnormalities (double hit).

At the time of ide-cel therapy, 12 patients (48%) had an ECOG of 0–1 while 13 patients (52%) had an ECOG ≥ 2. Only one patient (4%) had evidence of poor kidney function with a glomerular filtration rate < 30 mL/min/1.73 m^2^. Among 12 patients who received a bone marrow biopsy within 6 months of receiving ide-cel, 4 patients (33%) had evidence of high tumor burden, with ≥60% plasma cell involvement. Additionally, 10 patients (40%) had one or more sites of extramedullary disease at the time of CAR T-cell therapy.

Patients had received a median of six lines of therapy (range, 4–12) prior to ide-cel therapy, including autologous stem cell transplant in 100% of patients. Four patients (16%) had received prior BCMA-targeted therapy, all via clinical trial: one received CAR T-cell therapy, one received an antibody drug conjugate, one received a bispecific antibody, and one received CAR T-cell therapy followed by a bispecific antibody. All 25 patients (100%) had triple-class refractory disease while 22 patients (88%) had penta-refractory disease.

### 3.2. Safety Outcomes

The incidence of CRS among all 25 patients was 92%, with 1 patient (4%) experiencing grade 3 CRS. The incidence of ICANS was 12%, with one grade 3 event (4%) and one grade 4 event (4%) (Table 2). At the time of data cutoff, no patients had experienced any motorneurotoxicities or developed any secondary malignancies due to ide-cel.

All patients also experienced cytopenia, including grades 3–4 anemia in 40% of patients, grades 3–4 neutropenia in 96% of patients, and grades 3–4 thrombocytopenia in 48% of patients. Thirteen of twenty-five patients (52%) with any-grade neutropenia received granulocyte colony stimulating factor. Seven of twenty-four patients (29%) with any-grade thrombocytopenia received a thrombopoietin receptor agonist. Twenty-four patients (96%) had hypogammaglobulinemia (defined as an IgG level < 700 mg/dL) within 6 months of ide-cel therapy, with twenty patients (80%) receiving prophylactic intravenous immunoglobulin. Among all 25 patients, a total of 15 documented infectious events occurred within 6 months of CAR T-cell infusion, including eight upper respiratory infections, two enterocolitis events (including one case of clostridium difficile infection), two cases of bacteremia, two urinary tract infections, and one sepsis event. Grades 3–4 infectious events occurred in 3 of 25 patients (12%).

Nineteen patients had positive serum CMV IgG antibody pre-treatment indicating prior infection; nine (47%) cases of CMV viremia were observed and five patients received treatment with antivirals. Among the nine cases of CMV viremia, five patients (56%) had a peak polymerase chain reaction (PCR) level of <35 IU/mL while the remaining four patients (44%) had peak PCR levels up to 477, 1020, 1510, and 1520 IU/mL. Among the five patients who received treatment, antiviral therapy was initiated at PCR levels of <35, 135, 745, 1020, and 1510 IU/mL. The patient with a peak PCR level up to 1520 IU/mL required IV foscarnet therapy due to pancytopenia. No end-organ damage was observed among any patient who experienced CMV reactivation.

### 3.3. Efficacy Outcomes

At the time of data censoring, the ORR was 84%. Fourteen patients (56%) achieved a sCR, four patients (16%) achieved a VGPR, three patients (12%) achieved a PR, and four patients (16%) had PD (Table 3). Notably, serologic markers of three of the VGPR patients had normalized, but these patients did not undergo confirmatory bone marrow biopsy assessment.

**Figure 1:**
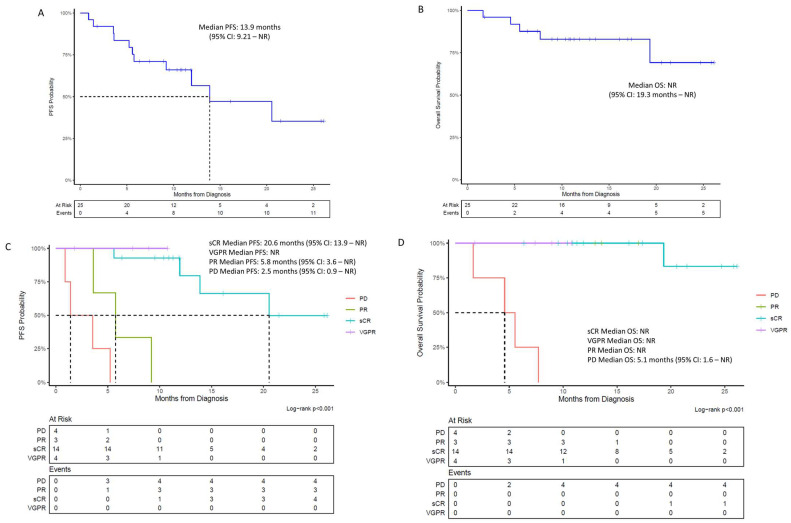
Kaplan–Meier survival curves. (**A**) Progression-free survival from date of ide-cel infusion to time of progression, relapse, or death. (**B**) Overall survival from date of ide-cel infusion to date of death. (**C**) Progression-free survival stratified by best overall response achieved. (**D**) Overall survival stratified by best overall response achieved.

The median follow-up time was 13 months. Median PFS was 13.9 months (95% confidence interval [CI]: 9.21—not reached [NR]) (Figure 1A). Among patients who had sCR, VGPR, PR, or PD, the corresponding PFS (95% CI) were 20.6 months (13.9—NR), not reached, 5.8 months (3.6—NR), and 2.5 months (0.9—NR), respectively (log-rank *p* < 0.0001) (Table 3, Figure 1C). PFS at 12 months was 57% (95% CI, 37–86%).

Median OS in all patients was not reached (95% CI: 19.3 months—NR) (Figure 1B). OS at 12 months was 83% (95% CI, 69–100%). Median OS for those who achieved any response was not reached compared to 5 months for those who never responded (95% CI: 1.6–NR) (log-rank *p* < 0.0001) (Table 3, Figure 1D).

At the time of data censoring, 5 of 25 patients (20%) had died. All five deaths were attributed to myeloma progression.

### 3.4. Factors Associated with Disease Relapse/Progression

Low magnitudes of correlation (absolute r < 0.25) were observed for all continuous variables (including age, ECOG, and number of prior therapies). Additional variables that were tested in the univariate and multivariate analyses included race, baseline ferritin, baseline C-reactive protein (CRP), baseline lactate dehydrogenase (LDH), high tumor burden (≥60% bone marrow plasma cells), extramedullary disease, high-risk cytogenetics, complex cytogenetics (defined as ≥3 chromosomal abnormalities), prior BCMA therapy received, and penta-refractory disease (triple-class refractory disease was not tested as all patients were triple-class refractory).

Univariate and multivariate analyses of all patients’ (n = 25) best ORR and PFS/OS, utilizing logistic and Cox regression models, respectively, did not demonstrate any significant predictors (Appendix A Table A1, Table A2 and Table A3).

### 3.5. Outcomes After CAR T-Cell Therapy Relapse

**Figure 2:**
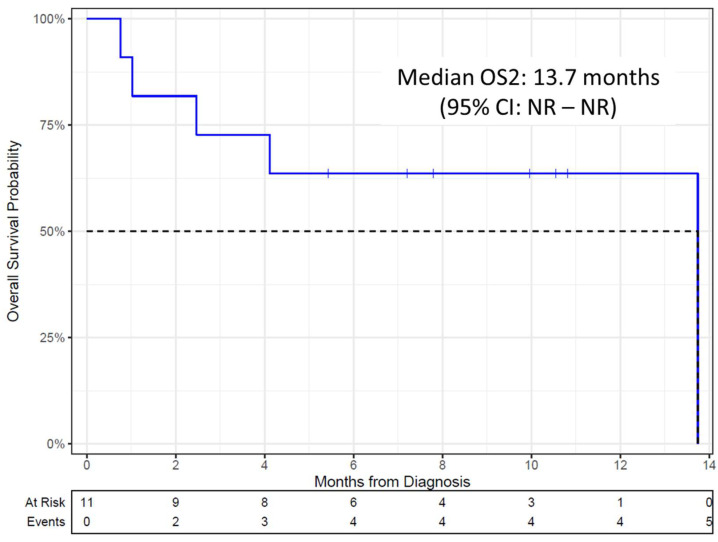
Kaplan–Meier estimate of OS2 from the date of relapse/progression after CAR T-cell therapy to the time of death.

Follow-up data were available for all 11 (44%) patients who had progressed or relapsed after ide-cel therapy. The median OS2 of these 11 patients was 13.7 months (95% CI: 4.11—NR) (Figure 2). The median OS2 in four patients who had no prior response to ide-cel was 1.7 months (95% CI: 0.76—NR) while that of seven patients who relapsed after achieving any response to ide-cel was 13.7 months (95% CI: NR—NR).

Patients received a median of one additional treatment after ide-cel therapy failure (range, 0–5) (Table 4). Six of eleven patients were alive at the time of data cutoff. Three patients were responding to teclistamab, two patients were responding to talquetamab, and one patient had only received local radiation therapy.

## 4. Discussion

Ide-cel, as the first BCMA-targeting CAR T-cell product approved for R/R MM, has shown promising results in both the clinical trial and real-world setting. Given the small number of real-world studies that have evaluated outcomes of ide-cel, the comparison of results from clinical trials to real-world data remains valuable for clinical management. At the same time, considering the current limitations of CAR T-cell products, it remains essential to identify biomarkers that may predict successful therapy performance. To do so, we first presented the safety and efficacy outcomes of those with R/R MM who were treated with ide-cel therapy at our institution. We then aimed to evaluate various factors that could be predictive of therapy response. Finally, we discussed the outcomes and subsequent treatment regimens of the patient subset who did not respond to ide-cel.

Our patient population in this study differed in some key baseline characteristics compared to the cohort in the phase 2 KarMMa-1 trial. Our patient population generally had a poorer performance status (ECOG ≥ 2 was 52% in the current study versus 2% in KarMMa-1). The proportion of patients who were penta-refractory was also higher in our study (88% versus 26% in KarMMA-1) [5]. These findings also persist when compared to the multicenter real-world study of ide-cel in 159 patients from the myeloma CAR-T consortium, in which 19% of patients had an ECOG ≥ 2 and 44% of patients were penta-refractory [15]. The percentage of patients in the current study with high-risk cytogenetics and extramedullary disease, as well as the median number of prior therapies were similar to other reports [15].

Safety outcomes appeared to be comparable between our study and the results presented in previous clinical trials. Any adverse effects of CRS and ICANS in our cohort were managed following the National Comprehensive Cancer Network (NCCN) guidelines [16]. The incidence of any-grade CRS and ICANS within our patient population was 92% and 12%, respectively. There was only one grade 3 CRS event, one grade 3 ICANS event, and one grade 4 ICANS event. These outcomes align with the CRS incidence (84%) and ICANS incidence (18%) reported in the KarMMa-1 trial [5]. The majority of CRS and ICANS cases were grades 1–2. Therefore, it appears that such side effects of ide-cel can be well managed even within a patient population with a poor performance status.

Incidence rates of other adverse effects were also similar. Grade 3 or higher cytopenias within 6 months of ide-cel therapy were seen in 40–96% of patients in our cohort, which is comparable with those seen in KarMMa-1 (52–89%) [5]. Meanwhile, the incidence of any-grade infection and serious infection (≥grade 3) within 6 months of ide-cel in our cohort was 44% and 12%, respectively, which is comparable to 69%/22% in KarMMA-1 [5]. The real-world retrospective studies have also reported any grade infectious complications to be 31–34% [15,17]. Notably, we did not determine if serious infections were treatment related in this retrospective study.

The incidence of CMV viremia, specifically, was not provided in either of the two major clinical trials and has not been extensively reported in other real-world studies, as well. One prospective study of CMV seropositive individuals treated with different CAR T-cell therapies found a reactivation rate of 43% among seven patients who received ide-cel [9]. In our study, 19 patients had a positive CMV IgG antibody titer prior to CAR T-cell therapy and were at risk of reactivation. Surprisingly, 9 of the 19 patients (47%) experienced CMV viremia within 6 months of ide-cel treatment. Five of these nine patients received preemptive therapy (which was initiated at PCR values ranging from <35–1510 IU/mL). None of the patients experienced end-organ damage. One patient with a peak PCR level of <35 IU/mL received oral valganciclovir for 2 weeks before discontinuation upon two negative PCR tests. Patients may have received early preemptive treatment when there was less experience with ide-cel administration. Early treatment may be beneficial when considering evidence that CMV IgG positive individuals can lose their viral immunity with lymphodepletion and ide-cel administration [18]. The importance of this potential loss of immunity as a mechanism contributing to CMV reactivation is further reflected in the high rate of hypogammaglobulinemia found in our cohort (96% of patients within 6 months of ide-cel infusion) [19]. Ultimately, though, the threshold at which treatment should be administered remains unclear.

Our ORR (84%) and sCR rate (56%) were comparable to those of KarMMa-1 (73%/33%) [5]. Among other real-world studies, ORRs have ranged from 75–84% and ≥CR rates have ranged from 42–44% [15,17].

After a median follow-up time of 13 months, which is much longer than that of other real-world studies (5.7–6.1 months) [15,17], median OS in our study was not reached. OS at 12 months was 83% (95% CI, 69–100%). Median OS in the KarMMA-1 trial was 19.4 months [5]. Meanwhile, our study’s median PFS was 13.9 months, compared to 8.8 months in KarMMa-1 [5]. Median OS and PFS reported in the Myeloma CAR T Consortium retrospective study was 19.4 months and 8.8 months, respectively [15].

At this time, it is still unclear which biological and/or clinical variables may best predict therapy outcomes. Within retrospective studies of patients with R/R MM who underwent ide-cel therapy, extramedullary disease, prior BCMA targeted therapy, elevated inflammatory markers (including ferritin and C-reactive protein), plasma cell leukemia, and t(4;14) have all been associated with worse PFS [20,21]. In subgroup analyses of ide-cel clinical trial data, elevated serum BCMA levels at baseline, IgG heavy chain, and elevated prothrombin time-international normalized ratio were negatively associated with achieving CR/sCR in KarMMa-1 [22]. Meanwhile, in KarMMa-3, lower levels of beta-2 microglobulin, LDH, and soluble BCMA characterized 94% of patients who had a longer PFS (>15.7 months) [23]. A separate biomarker study of ciltacabtagene autoleucel (cilta-cel), another anti-BCMA CAR T-cell therapy, found a high ORR yet numerically shorter PFS among those with high-risk cytogenetics, high tumor burden (≥60% bone marrow plasma cells), and baseline plasmacytomas [24,25]. On both our univariate and multivariate analysis, no significant predictors of ORR, PFS, or OS were identified, except a trend for ECOG (HR 3.77; 95% CI: 0.8–17.9; *p* = 0.095) for PFS.

Of note, within our cohort, four patients (16%) had been treated with BCMA therapy before receiving ide-cel. In their multicenter retrospective cohort study, Ferreri et al. reported that among 50 patients with prior BCMA exposure, a lower overall response rate, median duration of response, and median PFS were seen compared to those who had not received prior BCMA treatment [10]. Among our four patients, the median time interval in between the last administration of prior BCMA therapy and ide-cel was 9.9 months (range, 4.1–17.6 months). While prior BCMA therapy was not linked with inferior outcomes on our multivariate analysis (likely in the setting of a small sample size), one patient had sCR, one patient had PR, and two patients had PD. The PFS for the responders who achieved sCR and PR were 21.5 months and 3.6 months, respectively. A similar pattern of lower response rates has also been observed thus far with the use of cilta-cel in patients who had previously received noncellular anti-BCMA immunotherapy [26].

For those who relapse after ide-cel therapy, outcomes remain poor, given the lack of salvage therapy options that are both proven and effective. In a subgroup analysis of KarMMA-1 patients, Rodriguez-Otero and colleagues reported a median PFS2 (time from ide-cel to second disease progression or death) and OS (time from ide-cel to death) of 6.1 months and 24.8 months, respectively, among 68 patients whose disease progressed after ide-cel [7]. Meanwhile, in another retrospective study of 79 patients who relapsed after BCMA-directed CAR T-cell therapy, median OS2 (time from relapse/progression after ide-cel to death) was 17.9 months [27]. Similar to both these studies, our findings yielded a comparable median OS2 of 13.7 months among 11 patients who relapsed/progressed post–ide-cel. Potential factors that may hinder the long-term efficacy of ide-cel therapy in multiple myeloma include the loss of BCMA expression over time, high tumor burden resulting in CAR T-cell exhaustion, and the weaker in vivo killing effects of 4-1BB CAR T-cell products [28,29].

In their study, Rodriguez-Otero et al. also revealed that the choice of subsequent anti-myeloma treatment post-CAR T-cell therapy varied greatly, including dexamethasone, carfilzomib, other anti-BCMA agents (i.e., belantamab), and other novel therapeutics (i.e., feladilimab, an inducible T-cell co-stimulator) [7]. The majority of our patients who chose to pursue subsequent salvage therapy went on to receive another T-cell–engaging agent; three patients are currently responding to teclistamab, a BCMA-directed bispecific antibody, while 2 patients are responding to talquetamab, a GPRC5D-targeting bispecific antibody [30,31]. In the cohort described by Van Oekelen et al., the ORR for those who received T-cell–engaging therapies at any point after CAR T-cell relapse was 91.4% (32/35 patients) [27]. Thus, the proper sequencing of BCMA-directed therapies (including the use of novel T-cell engaging agents) in the setting of R/R MM warrants additional investigation.

This study has several potential limitations. The single-institution and retrospective nature of our study limited the sample size, statistical power, and ability for longer-term follow-up. Also, minimal residual disease testing was not conducted in any patient. Furthermore, not all patients had laboratory/cytogenetics data available to adequately determine baseline R-ISS staging. Similarly, not all patients received a repeat bone marrow biopsy after ide-cel to allow for comprehensive assessment of therapy response.

## 5. Conclusions

Here, we provide an in-depth report of patient characteristics and outcomes among those with R/R MM who received ide-cel therapy. Though our cohort included patients with heavily pre-treated disease, we demonstrate comparable response and toxicity rates to those of currently published large-scale clinical trials. Of note, we do report a significant rate of CMV reactivation that has not been well documented in the literature. Optimal clinical management of CMV reactivation remains unclear. We did not find any significant predictors of ORR, PFS, or OS within our cohort. Further research thus remains necessary to identify potential predictive factors of response to ide-cel therapy, which will improve patient selection for ide-cel therapy versus other salvage therapy options. Moreover, among non-responders to ide-cel therapy, OS2 was poor; the most commonly used treatment regimens in patients experiencing relapsed/refractory disease to ide-cel were teclistamab and talquetamab. The early response to such agents observed within our cohort, with the caveat of a small sample size, suggests that failure of ide-cel therapy should not preclude the subsequent trialing of other novel T-cell directed therapies.

## Figures and Tables

**Table 1:** Baseline characteristics of 25 R/R MM patients receiving ide-cel.

	All
Number of Patients	25
Age in Years, median (range)	65 (50–79)
<70, n (%)	19 (76)
≥70, n (%)	6 (24)
≥75, n(%)	2 (8)
Male, n (%)	12 (48)
R-ISS Stage *, n (%)	
I	2 (8)
II	6 (24)
III	2 (8)
Unknown	15 (60)
ECOG Score, n (%)	
0	2 (8)
1	10 (40)
≥2	13 (52)
GFR, n (%)	
<30 mL/min/1.73 m^2^	1 (4)
≥30 mL/min/1.73 m^2^	24 (96)
Pre-CAR T-Cell Labs, median (range)	
Ferritin (ng/mL)	290 (21–2153)
CRP (mg/dL)	0.31 (0.30–11.37)
LDH (U/L) **	178 (122–445)
≥60% Bone Marrow Plasma Cells (n = 12), n (%).	4 (33)
Extramedullary Disease, n (%)	10 (40)
Cytogenetic Abnormalities **^†^**, n (%)	
High-Risk Abnormalities	12 (48)
t(4;14)	3 (12)
t(14;16)	2 (8)
del(17p)/p53 mutation/monosomy 17	3 (12)
gain/amp 1q	6 (24)
double hit	2 (8)
Standard-Risk Abnormalities	9 (36)
Unknown Cytogenetics	4 (16)
Prior Lines of Therapy, median (range)	6 (4–12)
Prior Autologous Transplant, n (%)	25 (100)
Prior BCMA Therapy, n (%)	4 (16)
Triple-Refractory, n (%)	25 (100)
Penta-Refractory, n (%)	22 (88)

Abbreviations: BCMA, B-cell maturation antigen; del, deletion; CRP, C-reactive protein; ECOG, Eastern Cooperative Oncology Group; GFR, glomerular filtration rate; ide-cel, idecabtagene vicleucel; LDH, lactate dehydrogenase; R-ISS, revised international staging system; R/R MM, relapsed/refractory multiple myeloma; t, translocation. * The R-ISS disease stage at diagnosis was determined using cytogenetic abnormalities in conjunction with the baseline levels of albumin, beta-2 microglobulin, and serum lactate dehydrogenase. ** Reference range for LDH was 25–175 units/liter. **^†^** Present at any time prior to CAR T-cell therapy.

**Table 2:** Safety outcomes within 6 months of ide-cel administration.

Safety Outcome	All (%)	Grade 1–2 (%)	Grade 3–4 (%)
CRS (n = 25)	23 (92)	22 (88)	1 (4)
ICANS (n = 25)	3 (12)	1 (4)	2 (8)
Infection (n = 25) *	11 (44)	9 (36)	3 (12)
CMV Viremia (n = 23)	9 (39)	8 (35)	1 (4)
Anemia (n = 25)	25 (100)	15 (60)	10 (40)
Neutropenia (n = 25)	25 (100)	1 (4)	24 (96)
Thrombocytopenia (n = 25)	24 (96)	12 (48)	12 (48)

Abbreviations: CMV, cytomegalovirus; CRS, cytokine release syndrome; ICANS, immune effector cell-associated neurotoxicity syndrome. * CMV viremia excluded.

**Table 3:** Efficacy outcomes–response rates, PFS, OS.

Response	All (n = 25)
Overall response rate, n (%)	21 (84)
sCR	14 (56)
CR	0 (0)
VGPR	4 (16)
PR	3 (12)
MR	0 (0)
SD	0 (0)
PD	4 (16)
Median PFS, months (95% CI)	13.9 (9.21—NR)
sCR, n = 14	20.6 (13.9—NR)
VGPR, n = 4	NR
PR, n = 3	5.8 (3.6—NR)
PD, n = 4	2.5 (0.9—NR)
Median OS, months (95% CI)	NR
Responders (>PR)	NR
PD	5.0 (1.6—NR)

Abbreviations: CI, confidence interval; CR, complete response; MR, minimal response; NR, not reached; OS, overall survival; PD, progressive disease; PFS, progression-free survival; PR, partial response; sCR, stringent complete response; SD, stable disease; VGPR, very good partial response.

**Table 4:** Treatment regimens administered post–ide-cel therapy progression/relapse.

Treatment Regimen	Number of Patients
Teclistamab	4
Talquetamab	4
Lenalidomide	1
Cyclophosphamide + Bortezomib + Dexamethasone	1
Cyclophosphamide + Doxorubicin + Bortezomib	1
Hyperfractionated Cyclophosphamide	1
Autologous Stem Cell Transplant	1

## Data Availability

Research data that support the findings of this study are securely stored in an institutional repository and are available to share from the corresponding author upon reasonable request.

## Appendix A

**Table A1:** Univariate and multivariate logistic regression analysis to identify prognostic factors of ORR.

Variable	Univariate Analysis		Multivariate Analysis *	
	OR (95% CI)	*p*-Value	OR (95% CI)	*p*-Value
Age	1.01 (0.87–1.20)	0.9	—	—
Gender	0.30 (0.01–2.82)	0.3	—	—
Race	2.50 (0.25–25.2)	0.4	—	—
ECOG	0.30 (0.01–2.82)	0.3	—	—
Ferritin	1.00 (1.00–1.00)	0.3	—	—
CRP	0.92 (0.63–1.58)	0.7	—	—
LDH	1.00 (1.00–1.01)	0.8	—	—
≥60% Bone Marrow Plasma Cells	7.29 (0.31–1178.37)	0.22	—	—
High-Risk Cytogenetics	0.36 (0.03–3.86)	0.4	—	—
Complex Cytogenetics	0.67 (0.06–6.84)	0.7	—	—
Extramedullary Plasmacytoma	0.44 (0.02–4.16)	0.5	—	—
Prior Lines of Therapy	0.75 (0.45–1.22)	0.2	0.54 (0.14–1.13)	0.2
Prior BCMA Therapy	0.11 (0.01–1.24)	0.071	0.01 (0.00–0.59)	0.093
Penta-Refractory Disease	25,699,732 (0–NA)	>0.9	—	—

Abbreviations: BCMA, B-cell maturation antigen; ECOG, eastern cooperative oncology group; NA, not applicable; OR, odds ratio; ORR, objective response rate. * Variables for multivariate analysis were chosen based on backward selection criteria of a *p*-value ≤ 0.2 from univariate analysis.

**Table A2:** Univariate and multivariate Cox regression analysis to identify prognostic factors of PFS.

Variable	Univariate Analysis		Multivariate Analysis *	
	HR (95% CI)	*p*-Value	HR (95% CI)	*p*-Value
Age	0.96 (0.88–1.05)	0.4	—	—
Gender	1.98 (0.58–6.81)	0.3	—	—
Race	0.44 (0.13–1.47)	0.2	—	—
ECOG	3.77 (0.80–17.9)	0.095	4.78 (0.81–28.1)	0.084
Ferritin	1.00 (1.00–1.00)	0.13	1.00 (1.00–1.00)	0.3
CRP	1.06 (0.77–1.46)	0.7	—	—
LDH	1.00 (1.00–1.00)	0.8	—	—
≥60% Bone Marrow Plasma Cells	0.00 (0.00–∞)	>0.9	—	—
High-Risk Cytogenetics	1.86 (0.46–7.54)	0.4	—	—
Complex Cytogenetics	2.14 (0.51–9.06)	0.3	—	—
Extramedullary Plasmacytoma	0.55 (0.16–1.90)	0.3	—	—
Prior Lines of Therapy	1.00 (0.78–1.28)	>0.9	—	—
Prior BCMA Therapy	3.06 (0.78–12.1)	0.11	3.77 (0.48–29.4)	0.2
Penta-Refractory Disease	0.00 (0.00–∞)	>0.9	—	—

Abbreviations: HR, hazard ratio; PFS, progression-free survival. * Variables for multivariate analysis were chosen based on backward selection criteria of a *p*-value ≤ 0.2 from univariate analysis.

**Table A3:** Univariate and multivariate Cox regression analysis to identify prognostic factors of OS.

Variable	Univariate Analysis		Multivariate Analysis *	
	HR (95% CI)	*p*-Value	HR (95% CI)	*p*-Value
Age	1.01 (0.89–1.15)	0.8	—	—
Gender	1.64 (0.27–10.0)	0.6	—	—
Race	0.31 (0.05–1.85)	0.2	0.37 (0.05–2.96)	0.3
ECOG	3.05 (0.33–28.2)	0.3	—	—
Ferritin	1.00 (1.00–1.00)	0.2	—	—
CRP	1.00 (0.76–1.31)	>0.9	—	—
LDH	1.00 (1.00–1.00)	0.3	—	—
≥60% Bone Marrow Plasma Cells	0.00 (0.00–∞)	>0.9	—	—
High-Risk Cytogenetics	1.36 (0.22–8.19)	0.7	—	—
Complex Cytogenetics	1.11 (0.18–6.78)	>0.9	—	—
Extramedullary Plasmacytoma	1.11 (0.18–6.66)	>0.9	—	—
Prior Lines of Therapy	1.09 (0.78–1.53)	0.6	—	—
Prior BCMA Therapy	4.14 (0.69–25.0)	0.12	—	—
Penta-Refractory Disease	0.00 (0.00–∞)	>0.9	—	—

Abbreviations: OS, overall survival. * Variables for multivariate analysis were chosen based on backward selection criteria of a *p*-value ≤ 0.2 from univariate analysis.
